# Family history and risk of breast cancer: an analysis accounting for family structure

**DOI:** 10.1007/s10549-017-4325-2

**Published:** 2017-06-03

**Authors:** Hannah R. Brewer, Michael E. Jones, Minouk J. Schoemaker, Alan Ashworth, Anthony J. Swerdlow

**Affiliations:** 1 Division of Genetics and Epidemiology, The Institute of Cancer Research, London, SW7 3RP UK; 2 Division of Breast Cancer Research, The Institute of Cancer Research, London, SW7 3RP UK; 3 Breast Cancer Now Toby Robins Research Centre at The Institute of Cancer Research, London, SW7 3RP UK; 4 Division of Molecular Pathology, The Institute of Cancer Research, London, SW7 3RP UK; 5 UCSF Helen Diller Family Comprehensive Cancer Center, San Francisco, CA 94158 USA; 6 Division of Genetics and Epidemiology, The Institute of Cancer Research, Sir Richard Doll Building, Sutton, Surrey, SM2 5NG UK

**Keywords:** Breast cancer, Risk factors, Cohort study, Family history

## Abstract

**Purpose:**

Family history is an important risk factor for breast cancer incidence, but the parameters conventionally used to categorize it are based solely on numbers and/or ages of breast cancer cases in the family and take no account of the size and age-structure of the woman’s family.

**Methods:**

Using data from the Generations Study, a cohort of over 113,000 women from the general UK population, we analyzed breast cancer risk in relation to first-degree family history using a family history score (FHS) that takes account of the expected number of family cases based on the family’s age-structure and national cancer incidence rates.

**Results:**

Breast cancer risk increased significantly (*P*_trend_ < 0.0001) with greater FHS. There was a 3.5-fold (95% CI 2.56–4.79) range of risk between the lowest and highest FHS groups, whereas women who had two or more relatives with breast cancer, the strongest conventional familial risk factor, had a 2.5-fold (95% CI 1.83–3.47) increase in risk. Using likelihood ratio tests, the best model for determining breast cancer risk due to family history was that combining FHS and age of relative at diagnosis.

**Conclusions:**

A family history score based on expected as well as observed breast cancers in a family can give greater risk discrimination on breast cancer incidence than conventional parameters based solely on cases in affected relatives. Our modeling suggests that a yet stronger predictor of risk might be a combination of this score and age at diagnosis in relatives.

**Electronic supplementary material:**

The online version of this article (doi:10.1007/s10549-017-4325-2) contains supplementary material, which is available to authorized users.

## Introduction

Breast cancer is the most common cancer in women and the leading cause of cancer-related deaths in women worldwide [1]. In addition to well-established reproductive and lifestyle risk factors such as early age at menarche and HRT intake, there is a strong risk in relation to family history of breast cancer, with a twofold increase in risk of developing the disease for women with breast cancer in their first-degree family, and a larger increase in risk among women with a first-degree relative diagnosed before age 50 compared with after age 50 years [2–4].

In assessing risk of breast cancer, the categorization of family history as a risk factor for breast cancer has ranged from presence or absence of a family history [5–13] to more specific descriptions of cases in the family such as the number, type, age at diagnosis (e.g., ≤45 or >45 [14, 15], or ≤50 or >50 years [11, 14, 16]) of relatives who have had breast cancer [16–25], and a combination of the type of relative and age at diagnosis [3, 26]. These methods did not consider, however, the number of female relatives, and the person-years they have lived through, by age and calendar period, i.e., the denominator of the family’s risk. Women with many relatives who have reached older ages would be expected, for that reason alone, to have more relatives with breast cancer on average than those whose relatives are few and young. Cohort analyses of cardiovascular disease [26] and breast cancer mortality [27] have published risks in relation to a family history score that takes account of family structure. We therefore used data from the Generations Study, a cohort study of women in the UK, to analyze breast cancer incidence risks in relation to a family history score that takes account of the person-years at risk by age and calendar period, and the relatives’ ages at breast cancer incidence, and hence the numbers of breast cancers expected in the family.

## Methods

The Generations Study (GS) is a prospective cohort study that began recruiting women aged 16 or older from the general population of the United Kingdom in 2003 and now comprises over 113,000 women who completed an extensive questionnaire and provided consent [28]. The first follow-up questionnaire was sent to GS participants about two and a half years after their entry to the study and subsequent follow-up questionnaires at intervals of about three and a half years. The study was approved by the South East Multi-Centre Research Ethics Committee.

The current analytic cohort is based on women who joined the study between June 2003 and June 2012, inclusive. Participants who had been diagnosed with breast cancer before entering the study (*n* = 6604) or who reported that they did not know about their biological parents or siblings (*n* = 3905) were excluded. This left 103,738 participants who formed the analysis cohort. Breast cancers occurring in GS cohort participants were reported by these participants in their follow-up questionnaires, and later confirmed by cancer registry records, general practitioners, pathology records, or through ‘flagging’ at the National Health Service Central Registers (registers of England, Wales, and Scotland populations to which GS participants can be matched, and deaths and national cancer registrations then reported to authorized researchers).

### Statistical analysis

#### Assessment of breast cancer risk in families of participants

Information about first-degree relatives’ dates of birth, cancer history, and, for parents only, dates of death was provided by the participants in their recruitment questionnaire. There were 294,100 recorded first-degree female relatives of participants in this analysis cohort. For a small proportion of relatives, year of birth was not stated, or the stated year was impossible (*n* = 12,458, 4.2% of relatives), and for these women, year of birth was estimated (e.g., where birth year was unknown for mothers of participants, we assumed that the mother was older than the participant by the average age at childbirth in her generation). For analysis, we considered for each participant, all female full first-degree relatives as her ‘family cohort’ (i.e., half-sisters were excluded). Each relative in such a family cohort was considered to enter risk at her own date of birth and to leave risk at her year of breast cancer diagnosis, year of death (for mothers only), or the year the participant’s recruitment questionnaire was received (i.e., the last date information was provided about the relative), whichever occurred earliest.

We calculated the expected number of breast cancers in each family cohort by multiplying the cumulative person-years in the family cohort, stratified by age and calendar year, by the corresponding national annual, age-specific breast cancer incidence rates, and then summing across all strata. Published national breast cancer incidence rates for England and Wales combined were only available from 1971 to 1998. Rates from 1911 to 1970 were estimated by multiplying the age-specific breast cancer mortality rates in these years by the average age-specific ratio between published England and Wales incidence and mortality rates during 1971–1979. The average of the estimated incidence rates from 1911 to 1920 was used for years before 1911, when age-specific breast cancer mortality rates were not available. English national incidence rates were used for years after 1998 because combined England and Wales rates were not published after then. However, the difference will have been negligible since England contributes 94.6% of the combined population.

The total observed number of first primary breast cancers occurring in relatives in the family cohort was divided by the number of expected breast cancers, calculated as above, to produce a standardized incidence ratio (SIR) for that family. We will refer to this SIR as the ‘Family History Score’ (FHS).

#### Assessment of breast cancer risk in participants in relation to family history

We assessed risk of breast cancer in GS participants in relation to the FHS of their family, by calculating hazard ratios (HR) using Cox-proportional hazards regression with left truncation and right censoring, with age as the underlying time scale [29]. The FHS was ordered into six groups, and these groups were scored 0–5 for trend tests. We also similarly assessed breast cancer risk in GS participants in relation to presence of a family history of breast cancer (yes/no), the number of relatives with breast cancer (0,1, ≥2), the type of relative(s) with breast cancer (none, mother, sister, daughter, ≥2 relatives), and the age of the youngest relative with breast cancer (none, <45, ≥45 years). Relative risks of breast cancer were adjusted for age at menarche, benign breast disease, oral contraceptive use, parity, age at first birth, breastfeeding, age at menopause and menopausal status, hormone replacement therapy use, physical activity, pre- and post-menopausal body mass index, alcohol intake, smoking status, and socioeconomic status. For the HR calculations, participant entry to risk began on the date of completion of the recruitment questionnaire, and exit from risk was on the date the participant was diagnosed with breast cancer, date of last follow-up questionnaire, emigration, loss to follow-up, or death, whichever occurred earliest up to 30th August 2015.

To observe the impact of unknown vital status in sisters and daughters (i.e., of the assumption that they did not die before the other exit criteria had occurred), we conducted sensitivity analyses reducing the follow-up time (and hence expecteds) for sisters and daughters of GS participants in line with mortality rates in women of similar ages to the sisters and daughters without a history of breast cancer.

Because of the recruitment method [28], about 28% of participants have a first-degree relative who is also a GS participant, and in sensitivity analyses, we removed all participating relatives who joined the GS after the first participating relative.

For the main analyses, in situ diagnoses in participants were included together with invasive breast cancers, since ductal carcinoma in situ (DCIS) is widely considered to be a precursor of invasive breast cancer [30]. Women with such diagnoses are often treated with a mastectomy or lumpectomy and sometimes radiation and/or hormonal therapy [31]. Sensitivity analyses were also conducted, however, restricted to invasive breast cancers.

A likelihood ratio test was used to compare the contribution of the FHS to models with the presence of a family history of breast cancer (yes/no), the number of relatives with breast cancer (0.1, >2), the type of relative(s) with breast cancer (non, mother, sister, daughter, ≥2 relatives), and the age of the youngest relative with breast cancer (none, <45, ≥45), i.e., measures of family history used in previous epidemiological studies [5–26]. All statistical tests were two sided, and analyses were done using Stata (version 14.0) [31].

## Results

As of 30 August 2015, of the 103,738 GS participants, 1,474 were diagnosed with invasive breast cancer during follow-up and 259 with in situ diagnoses, giving a total of 1733 who reported breast cancer, with 99.8% confirmed from medical records. Follow-up questionnaires were completed by 96.3% of participants, and cancer status known from flagging for a further 1.7%. The remaining participants had either died (0.8%) or were lost to follow-up (1.2%). Total follow-up was 627,944 person-years, an average of 6.1 years per cohort member.

Descriptive characteristics of women in the GS cohort are shown in Table 1. Almost half of the participants were aged 45–64 years (47.4%) at recruitment, and 64.4% joined the study during 2006–2009. A slight majority (55.4%) of women who developed breast cancer during follow-up were diagnosed before age 60, with the overall mean age at diagnosis 53 years. There were 15,520 participants (15%) who reported one or more relative(s) with a history of breast cancer at recruitment, with most relatives (61.5%) diagnosed before age 60 and the overall mean age at diagnosis 57 years.

**Table 1:** Descriptive characteristics of the Generations Study cohort members in the United Kingdom and their family history of breast cancer

Characteristic	Number	Percent (%)
Participants in cohort		
Age at entry to the study (years)		
16–24	5591	5.4
25–34	17,430	16.8
35–44	22,872	22.0
45–54	24,668	23.8
55–64	24,459	23.6
65–102	8718	8.4
Total	103,738	100.0
Year of entry		
2003–2005	34,681	33.4
2006–2009	66,756	64.4
2010–2012	2301	2.2
Total	103,738	100.00
Age at breast cancer diagnosis (years)		
<30	7	0.4^*^
30–44	218	12.6
45–59	734	42.4
≥60	774	44.6
Total	1733	100.0
Family history of breast cancer (no. of affected relatives)		
0	88,219	85.0
1	14,750	14.2
2	669	0.7
≥3	100	0.1
Total	103,738	100.0
First-degree, female relatives of participants in cohort		
Age at breast cancer diagnosis (years)		
<30	149	0.9^a^
30–44	2679	17.3
45–59	6716	43.3
≥60	5976	38.5
Total	15,520	100.0

^*^Percentages among those with breast cancer

^a^Percentages among relatives with breast cancer

The relative risks of breast cancer in participants in relation to FHS are shown in Table 2. Eighty five percent of participants had no family member with breast cancer (i.e., FHS = 0), 8% had non-zero scores up to 20, and 7% had scores greater than this, with only 0.9% participants having a score ≥500. Risk of breast cancer increased significantly as the FHS increased (*P*_trend_ <0.0001). Participants in the highest FHS category had a relative risk of 3.50 (95% CI 2.56–4.79; *P* < 0.0001) compared with those with no affected relatives.

**Table 2:** Relative risks of breast cancer in Generations Study members by family history score (FHS), adjusted for other breast cancer risk factors^*^

Family History Score	No. of study participants	Person-years (1000)	No. of breast cancer cases	HR	95% CI	*P*
0^a^	88,219	534.5	1,301	1.00	baseline	
<10	3610	21.7	126	1.61	1.34, 1.94	<0.0001
10–<20	4563	27.5	134	1.63	1.36, 1.95	<0.0001
20–< 50	4334	26.2	94	1.72	1.39, 2.12	<0.0001
50–< 100	1692	10.2	37	2.11	1.52, 2.92	<0.0001
≥100	1320	7.9	41	3.50	2.56, 4.79	<0.0001
Total	103,738	627.9	1,733	Test for trend^b^: <0.0001		

*HR* Hazard Ratio from Cox regression using age as time scale, *CI* Confidence interval

^*^Adjusted for age at menarche, benign breast disease, oral contraceptive use, parity, age at first birth, breastfeeding, age at menopause, hormone replacement therapy use, physical activity, pre- and post-menopausal body mass index, alcohol intake, smoking status, and socioeconomic status

^a^No history of breast cancer in first-degree female relatives

^b^Test for trend across six groups scored 0–5

The analysis in Table 2 was also conducted for risk of estrogen receptor-positive and estrogen receptor-negative breast cancers separately (Supplement Tables 1 and 2, respectively); there was a similar increase in risk of each as the FHS increased (FHS ≥100 HR = 3.12 95% CI 2.14–4.55, *P*_trend_ < 0.0001; and FHS ≥ 100 HR = 3.61 95% CI 1.69–7.72, *P*_trend_ = 0.0001, respectively). In sensitivity analyses after reducing follow-up time in sisters and daughters without a history of breast cancer (see Methods), there was no change in relative risks of breast cancer (FHS ≥ 100 HR = 3.50, 95% CI 2.56–4.79, *P*_trend_ < 0.0001). When sensitivity analyses were conducted with only one participant proband included if more than one family member had joined the cohort (see Methods), there was still a similar increase in risk (FHS ≥ 100 HR = 3.31 95% CI 2.38, 4.60, *P*_trend_ < 0.0001). Analysis of invasive breast cancer only (*n* = 1474 cases) also showed a significant increasing trend (FHS ≥ 100 HR = 3.09, 95% CI 2.16–4.43, *P*_trend_ < 0.0001). Omitting participants for whom date of birth was missing for any relatives had no material effect on the results.

Table 3 shows the adjusted relative risks of breast cancer in the GS based on several other methods of family history categorization. The relative risk of breast cancer in women with at least one first-degree female relative with breast cancer was increased compared with those without a family history (HR = 1.77, 95% CI 1.58–1.97, *P* < 0.0001), while the breast cancer risk in participants with two or more relatives diagnosed with breast cancer more than doubled (HR = 2.52; 95% CI 1.83–3.47; *P* < 0.0001). About 5% of participants with two or more relatives with breast cancer (*n* = 41) fell into the highest FHS score group, 10% into the FHS 50 ≤ 100 group (*n* = 72), and 85% (*n* = 656) had a FHS below 50.

**Table 3:** Relative risks of breast cancer in Generations Study participants based on various commonly used categorizations of family history, adjusted for other breast cancer risk factors^*^

Breast cancer in family^a^	No. of study participants (%)	Person-years (1000)	No. of breast cancer cases	HR	95% CI	*P*
Diagnosis of breast cancer in relative						
No	88,219 (85.0)	534.5	1,301	1.00	baseline	
Yes	15,519 (15.0)	93.4	432	1.77	1.58, 1.97	<0.0001
No. of relatives with breast cancer						
0	88,219 (85.0)	534.5	1,301	1.00	baseline	
1	14,750 (14.2)	88.9	393	1.72	1.53, 1.92	<0.0001
≥2	769 (0.8)	4.5	39	2.52	1.83, 3.47	<0.0001
Type of relative with breast cancer						
Unaffected relative	88,219 (85.0)	534.5	1,301	1.00	baseline	
Mother only	11,940 (11.5)	71.9	295	1.72	1.51, 1.95	<0.0001
Sister only	2,730 (2.6)	16.5	96	1.73	1.40, 2.13	<0.0001
Daughter only	80 (0.1)	0.5	2	1.22	0.30, 4.89	0.78
Combination of relatives^a^	769 (0.8)	4.5	39	2.52	1.83, 3.47	<0.0001
Age of relative at diagnosis(years)^b^						
Unaffected relative	88,219 (85.0)	534.5	1301	1.00	baseline	
<45	2828 (2.8)	16.9	97	2.47	2.01, 3.04	<0.0001
≥45	12,691 (12.2)	76.5	335	1.63	1.45, 1.84	<0.0001

*HR* Hazard Ratio from Cox regression using age as time scale, *CI* Confidence Interval

^*^Adjusted for age at menarche, benign breast disease, oral contraceptive use, parity, age at first birth, breastfeeding, age at menopause, hormone replacement therapy use, physical activity, pre- and post-menopausal body mass index, alcohol intake, smoking status, and socioeconomic status

^a^First-degree female relatives

^b^Youngest if >1 relative with breast cancer

Breast cancer risks were similar in participants with a mother only (HR = 1.72; 95% CI 1.51–1.95; *P* < 0.0001) or sister only (HR = 1.73; 95% CI 1.40–2.13; *P* < 0.0001) with breast cancer. Participants who reported a relative diagnosed with breast cancer before age 45 had a relative risk of 2.47 (95% CI 2.01–3.04; *P* < 0.0001) which was significantly higher (*P* < 0.001) than those with an affected relative over age 45 (HR = 1.63 95% CI 1.45–1.84; *P* < 0.0001). None of these measures showed as great a risk discrimination as the FHS.

Likelihood ratio tests comparing models with and without the addition of the FHS to models with the measures of family history in Table 3 are shown in Table 4. The addition of the FHS gave a highly significant improvement to risk models containing binary family history (*P* < 0.001), the number of relatives with breast cancer (*P* = 0.001), the type of relative with breast cancer (*P* < 0.001), and the age of relative at breast cancer diagnosis (*P* = 0.01). Conversely, addition of binary family history or number of affected relatives to a model with FHS showed some evidence of significant improvement (*P* = 0.04 and *P* = 0.02, respectively), but the addition of type of relative to the FHS showed no significant improvement (*P* = 0.35). The best combination of variables was one with relative age at breast cancer diagnosis and FHS, for which the addition of either in the presence of the other showed a highly significant improvement (*P* = 0.006 and *P* = 0.01).

**Table 4:** Likelihood ratio test results comparing Cox-proportional hazards breast cancer risk models for different methods, and combinations of methods, of categorizing family history

Model	Goodness of fit		Likelihood ratio test^b^ ($\chi_{df}^{2}$)*, P* value
	*df*	*χ* ^2*^	
(a) Binary family history (Yes/No)	1	94.57	
(b) No. of relatives with breast cancer (0, 1, ≥ 2)	2	99.29	
(c) Type of relative with breast cancer (No, mother, sister, daughter, combination of relatives)	4	99.55	
(d) Age of relative at breast cancer diagnosis (No, < 45, ≥ 45)	2	106.41	
(e) Family History Score (FHS)^a^	1	102.42	
Combinations			
(a + e) Binary family history added to FHS	2	106.51	$\chi_{1}^{2}$ = 4.08, *P* = 0.04
(e + a) FHS added to Binary family history			$\chi_{1}^{2}$ = 11.94, *P* = 0.0005
(b + e) No. of relatives with breast cancer added to FHS	3	110.14	$\chi_{2}^{2}$ = 7.72, *P* = 0.02
(e + b) FHS added to No. of relatives with breast cancer			$\chi_{1}^{2}$ = 10.86, *P* = 0.001
(c + e) Type of relative with breast cancer added to FHS	5	112.74	$\chi_{4}^{2}$ = 10.31, *P* = 0.36
(e + c) FHS added to Type of relative with breast cancer			$\chi_{1}^{2}$ = 13.18, *P* = 0.0003
(d + e) Age of relative at breast cancer diagnosis added to FHS	3	112.63	$\chi_{2}^{2}$ = 10.20, *P* = 0.006
(e + d) FHS added to age of relative at breast cancer diagnosis			$\chi_{1}^{2}$ = 6.22*, P* = 0.013

^*^Variation accounted for by adding variables to model already including age at menarche, benign breast disease, oral contraceptive use, parity, age at first birth, breastfeeding, age at menopause, hormone replacement therapy use, physical activity, pre- and post-menopausal body mass index, alcohol intake, smoking status, and socioeconomic status

^a^Trend across six groups

^b^Test of improvement to the fit of the model by addition of alternative method for describing family history

## Discussion

Family history is an important breast cancer risk factor, and one that can cause considerable anxiety to women [32]. It is therefore important to measure the risk associated with it with as much discriminatory power as possible, both to improve overall risk prediction and for advice and information for women, especially those with affected relatives. Breast cancer incidence risk in relation to family history has been assessed in published studies by various parameters of the cases of breast cancer occurring in a woman’s first-degree relatives [3, 9, 11, 16, 17, 22, 33, 34]. However, it appears in principle that assessment of familial breast cancer risk should consider not only breast cancers observed in the family, but also the family size and age-structure and hence the expected number of cases if general population rates by age and calendar period prevailed in the family. Such analyses to divide risk by family history score have been undertaken for coronary heart disease and hypertension [26], and breast cancer mortality [27], and for all-cancer incidence in relatives of retinoblastoma patients [35]. To the best of our knowledge, such scores have not been calculated for breast cancer incidence, although one study compared risk in women dichotomized as with or without a family history, allowing for age but not calendar period expectations [36], and family structure has been taken into account when estimating risk of BRCA1 and BRCA2 status [37]. In our analysis using person-years based scores, the FHS discriminated risk more finely than measures based solely on breast cancer occurrence among relatives.

Because it is a continuous variable, the FHS allows for discrimination across the full spectrum of family histories in participants, while conventional discrete variables are confined to two or three categories of risk, with most of those with a positive family history falling into the lowest risk positive family history group (e.g., 393 participants in our study had 1 affected relative but only 39 had ≥2). The highest FHS group had a greater relative risk than any of the highest risk groups from conventional categorizations of family history. The majority of participants who had two or more relatives with breast cancer were not in the highest FHS group, but instead fell into other, lower FHS groups, reflecting that multiple affected family members may not indicate a very high risk if a woman comes from a very large family.

The addition of the FHS measure from Table 2 to models based solely on conventional aspects of cases in the family (yes/no; number of affected relatives; type of affected relatives, and age of relative at breast cancer diagnosis, as in Table 3) resulted in significant statistical improvements to the fit of the models. The combination of the age of relative at breast cancer diagnosis and FHS was the best fitted model. Although the age of a relative at breast cancer diagnosis is incorporated in the calculation of the expected number of cases (the denominator) in the FHS, it is not incorporated in the numerator (observed number of cases), unlike the metric of age at relative’s diagnosis on its own. Our study had insufficient cases for stable analysis of risk stratified by both FHS and age of relative at breast cancer diagnosis.

As with any observational study, there were some limitations. Reports of family history of breast cancer were provided by participants in questionnaires and were unconfirmed, but there is evidence that information from women reporting breast cancer in their first-degree relatives is likely to be highly accurate, with 99% specificity and 96% sensitivity reported [33, 38–40].

Another limitation was that vital status was only collected for parents in the baseline questionnaire. All sisters and daughters of participants therefore had to be considered alive, and those who had not been diagnosed with breast cancer were censored at the date the participant’s recruitment questionnaire was completed. For this reason, some family expected numbers are likely to be slightly overestimated, and subsequently the FHS slightly underestimated. Sensitivity analyses with reduced follow-up time for sisters and daughters, however, showed no material effect on the results.

As stated above, about 28% of participants had a first-degree relative who was also a GS participant, but after removing from analyses all participating relatives who joined the GS after the first participating relative (i.e., editing the cohort such that none of the participants are related to each other), results were essentially unchanged.

Most relatives of participants (70.3%) were born before 1971. Therefore, estimated incidence rates were used for some calendar years for the majority of relatives when calculating the expected number of family breast cancers, since data on national rates do not exist before 1971. However, many of the person-years before 1971 were at young ages when breast cancer is uncommon. Thus, any errors consequent on these national rate estimations are likely to have been slight, and anyway non-differential, and therefore unlikely to have influenced the relative risks materially. This applies more so to the estimates of national breast cancer mortality rates before 1911: only 3.8% of participants’ relatives were alive before 1911.

The FHS methodology could potentially be incorporated into risk prediction models for breast cancer, which currently use the number of first-degree relatives with breast cancer [14, 41–46]. The data used to calculate the FHS in first-degree female relatives are easily obtainable from women, making this measure suitable for employment in clinical settings, using a family score algorithm incorporating cancer registration rates. Finally, our modeling of breast cancer risks in relation to the FHS combined with other family history categorizations suggests that the best predictor of risk (if a sufficiently large dataset were available to validate it), might be a combination of FHS and age at diagnosis of breast cancers in relatives.

## Electronic supplementary material

Below is the link to the electronic supplementary material.

Supplementary material 1 (DOCX 19 kb)

## Abbreviations

FHS: Family history score
GS: Generations study
HR: Hazard ratio
CI: Confidence interval
SIR: Standard incidence ratio
DCIS: Ductal carcinoma in situ
