# Supplementary material for: Family history and risk of breast cancer: an analysis accounting for family structure
Source: Breast Cancer Res Treat. 2017 Jun 3;165(1):193–200. doi: 10.1007/s10549-017-4325-2 (PMC5511313; doi:10.1007/s10549-017-4325-2)
Supplement: Supplementary file 1 — Supplementary material 1 (DOCX 19 kb) [file 10549_2017_4325_MOESM1_ESM.docx]

**Supplementary Tables**

Supplement Table 1: Relative risk of estrogen receptor-positive breast cancer in Generations Study members stratified by Family History Score, adjusted for other breast cancer risk factors ^1^.

| Family History Score | No. of study members | Person-years (1000s) | No. of breast cancer cases | HR | 95% CI | *P* |
| --- | --- | --- | --- | --- | --- | --- |
| 0^2^ | 88,219 | 534.5 | 1,017 | 1.00 | baseline |  |
| < 10 | 3,619 | 21.7 | 98 | 1.59 | 1.29, 1.96 | <0.0001 |
| 10 - < 20 | 4,556 | 27.5 | 104 | 1.62 | 1.33, 1.99 | <0.0001 |
| 20 - < 50 | 4,332 | 26.2 | 80 | 1.90 | 1.51, 2.39 | <0.0001 |
| 50 - < 100 | 1,692 | 10.2 | 26 | 1.92 | 1.30, 2.83 | 0.001 |
| ≥ 100 | 1,320 | 7.9 | 28 | 3.12 | 2.14, 4.55 | <0.0001 |
| Total | 103,738 | 627.9 | 1,353 | Test for trend^3^: <0.0001 | | |
| HR: Hazard Ratio from Cox regression using age as time scale, CI: Confidence Interval  ^1^Adjusted for age at menarche, benign breast disease, oral contraceptive use, parity, age at first birth, breastfeeding, age at menopause, hormone replacement therapy use, physical activity, pre- and post-menopausal body mass index, alcohol intake, smoking status, and socioeconomic status.  ^2^No history of breast cancer in first degree female relatives.  ^3^Test for trend across six groups scored 0-5. | | | | | | |

Supplement Table 2: Relative risk of estrogen receptor-negative breast cancer in Generations Study members stratified by Family History Score, adjusted for other breast cancer risk factors ^1^.

| Family History Score | No. of study members | Person-years (1000s) | No. of breast cancer cases | HR | 95% CI | *P* |
| --- | --- | --- | --- | --- | --- | --- |
| 0^2^ | 88,219 | 534.5 | 197 | 1.00 | baseline |  |
| < 10 | 3,619 | 21.7 | 17 | 1.50 | 0.91, 2.48 | 0.11 |
| 10 - < 20 | 4,556 | 27.5 | 17 | 1.40 | 0.85, 2.29 | 0.19 |
| 20 - < 50 | 4,332 | 26.2 | 9 | 1.04 | 0.53, 2.02 | 0.92 |
| 50 - < 100 | 1,692 | 10.2 | 9 | 3.25 | 1.66, 6.38 | 0.0006 |
| ≥ 100 | 1,320 | 7.9 | 7 | 3.61 | 1.69, 7.72 | 0.0009 |
| Total | 103,738 | 627.9 | 256 | Test for trend^3^: 0.0001 | | |
| HR: Hazard Ratio from Cox regression using age as time scale, CI: Confidence Interval  ^1^Adjusted for age at menarche, benign breast disease, oral contraceptive use, parity, age at first birth, breastfeeding, age at menopause, hormone replacement therapy use, physical activity, pre- and post-menopausal body mass index, alcohol intake, smoking status, and socioeconomic status.  ^2^No history of breast cancer in first degree female relatives.  ^3^Test for trend across six groups scored 0-5. | | | | | | |
